# Pacing strategies in marathons: A systematic review

**DOI:** 10.1016/j.heliyon.2024.e36760

**Published:** 2024-08-23

**Authors:** Jungong Sha, Qing Yi, Xin Jiang, Zhengwei Wang, Houwen Cao, Shan Jiang

**Affiliations:** a School of Physical Education, Shanghai University of Sport, Shanghai, China; b College of Physical Education, Dalian University, Dalian, China; c Department of physical education, Dalian Jiaotong University, Dalian, China; d School of Kinesiology and Health Promotion, Dalian University of Technology, Dalian, China; e Department of Sports Science and Physical Education, The Chinese University of Hong Kong, Shatin, Hong Kong

**Keywords:** Pacing strategy, Marathoner, Running performance

## Abstract

**Background:**

The pacing strategy embodies the tactical behavior of athletes in distributing their energy across different segments of a race; therefore, a quantitative analysis of pacing strategies in marathon races could deepen the understanding of both pacing behavior and physical capacity in marathon athletics.

**Objective:**

The objective of this systematic review was to synthesize and characterize pacing strategies in marathon road races by exploring the categories of pacing strategies and the factors that influence these strategies during marathon events.

**Methods:**

Preferred Reporting Items for Systematic Reviews guidelines were followed for systematic searches, appraisals, and syntheses of literature on this topic. Electronic databases such as Science Direct, SPORTDiscuss, PubMed, and Web of Science were searched up to July 2024. Records were eligible if they included pace performance measurements during competition, without experimental intervention that may influence their pace, in healthy, adult athletes at any level.

**Results:**

A total of 39 studies were included in the review. Twenty-nine were observational studies, and 10 were experimental (randomized controlled trials). The assessment of article quality revealed an overall median NOS score of 8 (range 5–9). The included studies examined the pacing profiles of master athletes and finishers in half-marathon (n = 7, plus numbers compared to full marathon), full-marathon (n = 21), and ultramarathon (n = 11) road races. Considering that some studies refer to multiple pacing strategies, in general, 5 studies (∼13 %) reported even pacing, 3 (∼8 %) reported parabolic pacing, 7 (∼18 %) reported negative pacing, and 30 (∼77 %) reported positive pacing during marathon competitions. Gender, age, performance, pack, and physiological and psychological factors influence pacing strategies.

**Conclusion:**

This study synthesized pacing performance in marathons and highlighted the significance of examining pacing strategies in these events, offering valuable insights for coaches and athletes. Several key findings were highlighted: (1) pacing profiles and pacing ranges were identified as the primary indicators of pacing strategies; (2) the pacing strategy was found to be dynamic, with the most substantial effects attributed to gender and distance; and (3) three distinct types of pacing strategies for marathons were classified: positive, negative, and even pacing. These findings advance the understanding of marathon pacing strategies by shedding light on the factors that influence athletes’ pacing decisions and behaviors. Additionally, these findings offer practical benefits, aiding athletes in making well-informed tactical choices and developing effective pace plans to enhance marathon performance. However, due to the complex nature of marathon racing, further research is required to explore additional factors that might impact pacing strategies. A better grasp of optimal pacing strategies will foster progress in this area and serve as a basis for future research and advancements.

## 1 Introduction

Pacing denotes the distribution of speed throughout a process of motion [1]; it is typically expressed as the time needed to cover a given distance and is commonly utilized in long-distance timed competitive sports, such as marathons, swimming, and road cycling. In these timed events, the goal is to complete a known distance in as short a time as possible [2,3]. Therefore, pacing is the basic way to measure how quickly an athlete finishes a race and whether endurance is excellent [4].

Pacing strategy refers to the tactical approach of an athlete who manages energy distribution throughout different segments of a race to minimize speed reduction and maintain a steady pace. The essence of a pacing strategy involves the consistent maintenance of a specific speed cadence and relies on ongoing decision-making [5]. This type of decision-making is generally performed during a race, with adjustments made to pacing to minimize time loss based on fitness status and changing circumstances [6].

The rhythmic behavior of maintaining a specific pace at different stages varies according to the type of sport, the event duration, the athlete's knowledge and experience, and the competitive ability of opponents [7]. Most existing research on pacing strategies has concentrated on swimming, cycling, rowing, and skiing, with relatively little focus on running due to the limited availability of high-resolution official performance data prior to Abbiss and Laursen's work in 2008, which characterized pacing profiles for events at various distances [8]. They classified pacing strategies into six types: negative (increasing speed throughout the race), positive (decreasing speed throughout the race), “all-out” (initial rapid acceleration followed by a decrease in speed), even, parabolic (including u-, j-, and inverse j-types), and variable pacing characteristics. Since this review, new literature on pacing strategies has emerged, and these strategies are increasingly being applied to marathon events.

The marathon, as a popular sporting event, is favored and popular worldwide and requires athletes to have excellent physical fitness and endurance performance; therefore, the investigation of long-distance athlete pacing strategies to improve athletic performance is currently a significant topic in the academic world. The current definition of pacing strategy used by scholars in marathon programs is not consistent, and some scholars also understand it to include a variety of factors, such as pacing performance, pacing pattern, running tactics, pace control, and pacing profile [[9], [10], [11], [12], [13], [14], [15], [16], [17]]. Determining the appropriate pace for athletes should be guided by the existing peer-reviewed literature. Previous reviews have addressed pacing strategies in marathon races [1,18]. However, these studies did not provide a comprehensive and systematic analysis of pacing strategies; they only outlined and described the pacing performance and tactical behavior of elite athletes in specific races. Unlike other sports, such as swimming and skiing, which have systematic reviews of pacing strategies for various events, such reviews are lacking for marathon racing [[19], [20], [21]]. Therefore, the purpose of this review is to provide an overview of current research on pacing strategies in marathons and to investigate and summarize (1) the measurement and classification of pacing strategies in marathon events. (2) Factors associated with the influence of pacing strategies during marathons.

## 2 Methods

### 2.1 Search strategy

A thorough and methodical literature review was performed following the Preferred Reporting Items for Systematic Reviews and Meta-Analyses (PRISMA) guidelines [10]. This search included relevant studies available up to July 2024. Electronic databases were utilized to locate publications that implemented pacing strategies in sport science, focusing solely on studies conducted with healthy adult sports populations. Searches were conducted in three databases: ScienceDirect via Elsevier, PubMed, SPORTDiscuss via EBSCOHost, and Web of Science, starting from their inception. The search terms used were “Pace” OR “pacing” OR “Pacing strategy” OR “running performance” OR “tactical behavior” OR “velocity profile”. These terms were combined (AND) with “marathon” OR “long distance running” and combined (NOT) “cross-country” OR “trail”.

### 2.2 Study selection

Publications retrieved were initially screened by title to eliminate duplicates and those not relevant to the research question. Abstracts of the remaining studies were screened next, resulting in 39 studies being chosen for full-text assessment based on specific inclusion and exclusion criteria.

### 2.3 Inclusion and exclusion criteria

**Fig. 1:**
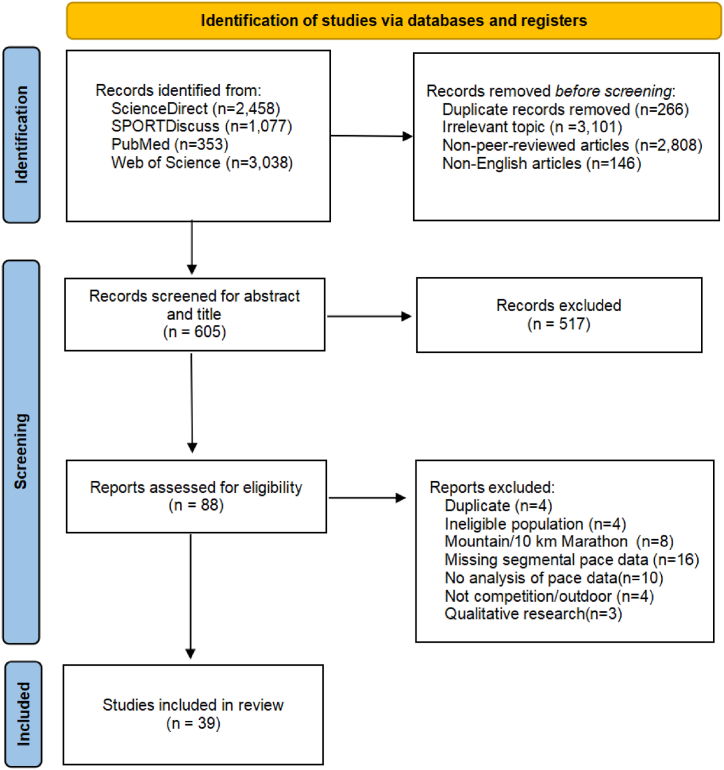
Preferred Reporting Items for Systematic reviews and Meta-Analyses (PRISMA) flow diagram of search strategy PRISMA illustrating the systematic review process and the inclusion and exclusion of research papers.

The inclusion criteria for selecting articles for the review were: (1) studies conducted in formats used in Olympic competitions (i.e., half marathon and full marathon), (2) segmental pacing data (e.g., comparisons between laps, various sections of the same race), and (3) actual international marathon events worldwide. Some consideration was also given to ultralong-distance races (e.g., ultramarathons over 50 km) and studies simulating marathon races outdoors. Exclusion criteria included (1) review articles, congress abstracts, editorials, or other non-original articles; (2) articles published in languages other than English; (3) studies mentioning mountains, cross-country, trail marathons, or 10 km runs; (4) studies involving participants under 20 years old (IAAF Marathon Age Limit) or with health conditions such as disease; or (5) studies that conducted qualitative analyses. In total, 39 studies were included for qualitative synthesis. The process of study selection and reasons for exclusion are depicted in Fig. 1.

Two independent reviewers, XJ and ZW, assessed the studies for inclusion, with any disagreements resolved through discussion and arbitration by HC if necessary. The reviewers, XJ and ZW, were aware of the authors, institutions, and journals of the publications. If a decision on the inclusion or exclusion of a paper could not be determined from the title and abstract alone, the full text was retrieved and evaluated.

### 2.4 Data extraction

For all selected studies, the following data were extracted: (i) author name; (ii) publication date; (iii) participant characteristics; (iv) study design; (v) pace measured; and (vi) pacing profile/pace range. These outcomes were documented for the narrative review.

### 2.5 Quality assessment

The methodological quality of the included studies was assessed by two reviewers (XJ and ZW) using the Newcastle–Ottawa Scale (NOS) for Cohort Studies, with specific customizations made to meet the requirements of this review [22]. The NOS, a recognized scientific assessment tool [34], is identified by the Cochrane Handbook for Systematic Reviews of Interventions as one of two beneficial tools and permits customization relevant to the research question [35]. Each study was evaluated across three domains: selection of the study groups; comparability of cohorts based on design and analysis; and outcome, including adequate follow-up for the occurrence of the outcome. Maximum scores of 4, 2, and 3 were assigned to each respective area, yielding a total possible score of 9. The selection process for the study groups was evaluated for its representativeness of the exposed and non-exposed cohorts, ascertainment of exposure, and verification that the outcome of interest was absent at the start of the study. Cohort comparability was assessed, focusing on the design factors controlled by the study. Outcomes were evaluated in terms of assessment methods, follow-up duration, and adequacy of follow-up for the cohort. The NOS was chosen for continuity, as it has been employed in numerous previous systematic reviews on sport topics [[23], [24], [25]], and was deemed the most suitable quality assessment tool for these analyses.

### 2.6 Data synthesis

Qualitative analysis was conducted on the selected studies, focusing on the associations between pacing strategies, characteristics of marathon athletes, and pacing performance. A narrative review was presented in the text, and tables summarize the characteristics of the studies.

## 3 Results

Of the 2458 (ScienceDirect), 1077 (SPORTDiscuss), 353 (PubMed), and 3038 (Web of Science) articles retrieved, 3367 were excluded due to title duplications (266) or irrelevant topics (3,101). An additional 2954 records were also excluded for not being peer-reviewed in English. From the remaining 605, a further 517 records were excluded after titles and abstracts were screened. Forty-nine studies were excluded from the remaining 88 articles. Consequently, 39 studies were retained for inclusion in the final stage of this review.

For each article, a standardized document form was utilized to extract relevant information from the selected papers, including the category of the marathon, authors, participants, research design, analyzed variables, segment types, indicators of pace, pacing profiles, and a summary of the main findings (Table 1).

**Table 1:** Descriptive results and characteristics of included studies.

Study	Sample	Research design	Categories	Variables	Segment	Pacing profiles	Main results
Cuk et al. [26]	17,465 finishers in 2017 Vienna City Marathon	Descriptive	Age (2 groups), Race (2 groups)	Average speed from each segments, Pace range, End spurt	5 km-5 splits (half marathon) 10 km-5 splits (full marathon)	Positive	Marathon runners showed greater variability in pacing than half-marathon runners.Women showed no differences in pace variability in regard to the age group.Younger half-marathon men and women showed the fastest end spurt compared to older age groups and marathon runners.
Louis et al. [36]	2 runners who broke the World Record time for combined father and son in 2019 Frankfurt Marathon	Quasi-Experimental	NR	physiological profiles, Average speed from each segments, CV of speed	5 km-9 splits	Even (father) Positive (son)	Father and son who broke the World record time for combined father and son marathon had a similar level of performance, but their physiological profiles and pacing strategies during the marathon were different.
Sengoku et al. [61]	2 experienced ultra-marathon runners	Quasi-Experimental	NR	blood glucose fluctuation, Average speed	5 km-20 splits	Positive	Fluctuations in blood glucose levels are not related to pacing strategy
Angus et al. [37]	2 runners who create the most recent world-record marathon runs	Descriptive	NR	Headwind Gradient Deviations in average speed from each segments	1 km-43 splits	U-shaped (Runner 1) Variable (Runner 2)	Headwind was a significant factor in running speed variability for both runners, with Runner 2 targeting the (optimal) parallel variation principle, whilst Runner 1 did not. After adjusting for these responses, neither runner followed the (optimal) ‘even’ power pacing principle.
Muñoz-Pe′rez et al. [43]	2295 runners in 2017 Berlin marathon	Descriptive	Performance Level (4 groups)	Average speed from each segments, Dif-half	5 km-10 splits	Even and positive	Elite and trained male marathoners used similar pacing strategies, whereas female athletes' pacing strategies varied significantly across levels; athletes mainly used an even pacing strategy and secondarily used a positive pacing strategy.
Nikolaidis et al. [38]	298,082 finishers in 2006–2016 New York City marathon	Descriptive	Performance Level (4 groups)	Average speed from each segments, Deviations in average speed from each segments, End Spurt	5 km-9 splits	Positive	The slowest group had the largest percentage decrease in speed at 5, 10, 15, and 20 km but the largest percentage increase in speed at 35 and 40 km,the fastest group had the least decrease during the race and the least increase at 40 km.Age affects the change in pace, but this effect becomes smaller as the level of performance increases
Oficial-Casado et al. [44]	91,493 finishers from four different races (Valencia, Chicago, London and Tokyo Marathon)	Descriptive	Performance Level (6 groups), Location (4 groups)	Average speed from each segments, Pace range,Dif-Half, Percentage of the mean speed in full race from each segments	5 km-9 splits	Positive	race characteristics affect pacing, being this effect higher as the category time increases. Although races did not differ at each section for high performance runners, Valencia marathon had values closer to a negative pacing profile than London marathon.
Trubee et al. [45]	36,223 finishers in 2007 and 2009 Chicago Marathon	Descriptive	Age(3 groups), Performance Level (2 groups), Heat stress (2 groups), Sex(2 groups)	Average speed from each segments, Percentage change between 30 km and last 12.2 km	5 km-9 splits	Positive	Age, sex, heat stress and performance all affect pacing changes; non-elite females can maintain speed better than males, temperature amplifies sex differences, no difference in pacing changes in elite groups; slower initial speeds should be used in hotter temperatures in order to maintain or improve pace in later stages
Arie-Willem et al. [17]	98,563 finishers in 2015–2017 Boston Athletic Association	Descriptive	Race (3 groups)	Percentage of the mean speed in full race from each segments	5 km-9 splits	Positive(full), Negative and positive(half), Even(10 km)	Pacing has a large impact on the result in long-distance running event.The profile with the smallest speed drop throughout the race is the most dominant pacing profile among the group of fast athletes.
Arturo et al. [57]	20 world record from 1998 to 2023, 28 champion of marathon World Championship or Olympic Games from 2001 to 2023	Descriptive	Race(2 groups), Sex(2 groups)	Average speed from each segments, Endspurt, CV of speed, Percentage of the mean speed in full race from each segments,	5 km-9 splits	Positive(man), Even(women)	Marathon WRs are characterized by fast, even and sustained paces, slower paces and more negative pacing approaches with fast endspurts are adopted during winning major championship performances.

Study	Sample	Research design	Categories	Variables	Segment	Pacing profiles	Main results
Matta et al. [53]	10 participants in two 6-h ultra-marathon races with two pacing strategies	Quasi-Experimental	Pacing(2 groups)	Critical speed, Percentage of the mean speed in full race from each segments	36 min-10 splits	Positive	Slow starts do not affect an athlete's overall pacing strategy in an ultra-marathon race
JoJo et al. [46]	15 marathon world records for men during 1967 and 2014	Descriptive	Era(Classic vs Contemporary)	Average speed from each segments, Percentage of the mean speed in full race from each segments, CV of speed	5 km-9 splits	Positive(classic) Negative(Contemporary)	The pacing strategies of the best marathon runners in the world have changed over the last 50 years. Although a negative pace distribution has been proposed as the most efficient option, a pacing strategy characterised by very little speed changes across the whole race may be the way to go in the future.
Andrew et al. [47]	196 finishers in a 100-km race incorporating the World Masters Championships	Descriptive	Performance Level (4 groups), Age(5 groups), Sex(2 groups)	Average speed from each segments, Percentage of the mean speed in full race from each segments, CV of speed	10 km-10 splits	Positive	Females showed lower relative starting speeds and higher finishing speeds than males.Although pacing remained consistent across age categories, it differed with level of performance within each. Strategy differs between genders and differs depending on performance level achieved in males but not females.
Santos-Lozano et al. [48]	190,228 finishers in New York City marathon from 2006 to 2011	Descriptive	Performance Level (4 groups), Sex(2 groups)	Average speed from each segments, ACSS,CV of speed	5 km-9 splits	Positive	Although all runners developed a positive pace profile, a lower variability of speed through the race was found in the top runners compared with the less successful runners.
Breen et al. [41]	31,762 masters athletes from the 2015 TSC New York City Marathon	Descriptive	Performance Level (7 groups), Age(10 groups), Sex(2 groups)	Average speed from each segments, Pace Range, End Spurt	5 km-9 splits	Controlled(Pace Range)	High performing masters athletes use more controlled pacing strategies, compared to their lower ranked counterparts, independent of age and gender.
Beal et al. [31]	58 elite runners from 2019 Doha and 2017 London IAAF World Athletics Championship	Descriptive	Performance Level (4 groups)	Average speed from each segments	5 km-9 splits (London) 1 km-43 splits (Doha)	Negative(faster), Positive(slower)	Elite athletes undertaking a marathon in extreme heat and humidity there was reduced performance, progressive slowing of pace and high-non completion rate.The faster athletes adopted a more conservative initial pacing strategy, lower athletes adopted a more ambitious earlier exercise pace unsustainable.
JoJo et al. [49]	18 marathon world records for men and women during 1998 and 2018	Descriptive	Sex(2 groups)	Average speed from each segments, Percentage of the mean speed in full race from each segments, CV of speed	5 km-9 splits	Positive(men)	Men and women have used different strategies when breaking world marathon records. While male athletes increased speed as the race progressed (negative strategy), female athletes used a less uniform pacing throughout the competition.
Knechtle et al. [33]	A recreational master runner from the 13th Festival Athens 48h Ultramarathon in 2018	Quasi-Experimental	NR	Body composition, Average speed from each segments	6 h-8 splits/1 km(1 lap)-230 splits	Positive	In a master runner competing in a 48 h run, running speed decreased non-linearly during the race as a positive pacing. While body mass decreased, body water increased.
Knechtle et al. [34]	A 95 years old European marathon record holder in the Sri Chinmony 12 + 24h Ultramarathon	Quasi-Experimental	NR	Body composition, Average speed from each segments	1104.4m(1 lap)-48 splits	Parabolic(U-shaped)	A 95-year-old man was able to run during 12 h using a U-shaped pacing and achieving a total distance of nearly 53 km. Increased selected hematological and biochemical parameters returned to pre-race values within a post-race recovery phase of 5 days.
Nikolaidis et al. [50]	48,565 finishers in the 2015 New York City marathon	Descriptive	Sex(2 groups), Age(14 groups)	Average speed from each segments, Percentage change between end split and first split	5 km-9 splits	positive	Men and women of all age groups reduced running speed during the marathon with a final spurt in the last segment. The speed decreased more in men compared to women with a different trend in the 25–30 km split(speed increased in women and decreased in men).

Study	Sample	Research design	Categories	Variables	Segment	Pacing profiles	Main results
Nikolaidis et al. [27]	9111 finishers in 2017 Ljubljana marathon	Descriptive	Sex(2 groups), Race (2 groups)	Percentage of the mean speed in full race from each segments, ACSS	5 km-5 splits (half marathon) 10 km-5 splits (full marathon)	Positive	Both half-marathon and marathon races presented a positive pacing.Half-marathon runners did not show an end spurt, which was observed in marathon runners. Women and men adopted similar pacing pattern in half-marathon, whereas in marathon, women had more even pacing than men.
Brian Hanley [9]	835 finishers in IAAF World Half Marathon Championships (2007–2010、2012、2014)	Descriptive	Performance Level (5 groups), Pack(6 groups)	Average speed from each segments, Dif-Half	5 km-5 splits	Positive	The best athletes displayed patterns of even pacing for some of the race, while slower athletes generally slowed from a peak speed at 5 km until they reached 20 km. All groups increased pace considerably during the final 1.1 km.
Nikolaidis et al. [42]	156 finishers in 2017 Athens Authentic Marathon	Quasi-Experimental	Performance Level (Male 4,Female 2), Age(M 8,F 2), Sex(2 groups)	Physiological characteristics, Psychological characteristics, Average speed from each segments, Pace Range, CV of speed	5 km-9 splits	Positive	Women, older and faster recreational runners adopted a more even pacing, it was related with a higher aerobic capacity and lower muscle strength in men.Men with more even pacing scored higher in psychological coping,self-esteem, life meaning, recognition and competition.
Aschmann et al. [40]	298,082 finishers in the New York City marathon (2006–2016)	Descriptive	Nationality (16 groups)	Average speed from each segments, Deviations in average speed from each segments,	5 km-9 splits	Even	Nationality may influence pacing.Ethiopians and Kenyans adopt a relatively more even pace during the New York city Marathon which is shown by their small changes in race speed and by practicing the end spurt less than other nationalities.
Bossi et al. [55]	501 finishers in the Rio 24h Ultra-marathon (2008–2012)	Descriptive	Performance Level (4 groups), Age(6 groups)	Average speed from each segments, Percentage of the mean speed in full race from each segments	1 h-24 splits	Parabolic(reverse J-shaped)	All runners displayed a rough, reverse J-shaped pacing strategy. They reduced speed during most of the race but slightly increased in the final hours; except in the very last one, when they reduced speed again. The best runners revealed a more conservative pacing strategy in the first hours compared with their slower counterparts.
Takayama et al. [51]	48 male finishers in the 2014 JinguGaien 24h Ultra-marathon	Descriptive	Performance Level (5 groups)	Average speed from each segments, Percentage of the mean speed in full race from each segments, CV of speed	1 h-24 splits	Positive	The faster runners ran at a relatively constant speed during the second half of the race when compared with the second-half speed of the slower runners.CV and total distance were significantly negatively correlated.
Beat Knechtle et al. [58]	1100 male elite runners from 100 km Lauf Biel in Switzerland (2000–2009)	Descriptive	Performance Level (10 groups), Age(10 groups), Edition(10 groups)	Average speed from each segments, Percentage of the mean speed in full race from each segments	Each segment-4 splits	Positive	Running speed decreased over segments in the top ten athletes in each edition, but remained unchanged over last segment.Runners in the age(18–24)showed the greatest decrease in running speed over the different segments.
Brian Hanley [52]	1222 elite runners from Doha IAAF World Athletics Championship(2001–2015) and Olympics Game in 2012	Descriptive	Performance Level (3 groups), Packing(6 groups), Sex(2 groups)	Average speed from each segments,Dif-Half, Percentage of the mean speed in full race from each segments,	5 km-9 splits	Positive, Negative (female)	Running with the same athletes throughout the race was conducive to achieving even pacing. Women were better at pacing than men, with fewer dropouts, less deceleration in the second half, and more negative splits.
Nikolaidis et al. [28]	9111 finishers in the 2017 Ljubljana Marathon	Descriptive	Age(8 groups), Sex(2 groups), Race(2 groups)	Average speed from each segments, Pace Range, End Spurt	5 km-5 splits (half marathon) 10 km-5 splits (full marathon)	Positive	All age groups presented a positive pacing in both race distances and genders. All groups showed an end spurt in the marathon,but not in the half-marathon. Most age groups in both genders exhibited a more even pace in the half-marathon than in the marathon.
Muñoz-Pérez et al. [60]	279 finishers in Berlin's Marathon from 2008 to 2018	Descriptive	Performance Level (5 groups), Packing(5 groups)	Average speed from each segments, DifHalf	5 km-9 splits	Positive	Positive pacing behaviors were predominantly followed by elite male marathon runners. The latest stages of the race were usually run alone in men and women and were also characterized by an accentuated decrease of pace and the generation of significant pace differences between performance groups at every distance segment.

Study	Sample	Research design	Categories	Variables	Segment	Pacing profiles	Main results
Billat et al. [54]	4 marathon world records from Berlin 2018,London 2019,Chicago 2019 and London 2003	Descriptive	Sex(2 groups)	Average speed from each segments, Critical Speed, Percentage of the mean speed in full race from each segments, CV of speed	1 km-43 splits	Negative	The best female and male marathon performances were run differently, and fractional use of CS.Improvement in marathon performance might depend on negative split and asymmetry for female runners, and on higher fractional utilization of CS for male runners.
Takayama et al. [35]	A 32 yr ultra-marathon runners in the Hirosaki 24h Ultra-marathon	Quasi-Experimental	NR	Blood glucose level, Heart Rate, Average speed from each segments, Critical Speed	1 h-24 splits	Positive	The runner's speed decreased during the middle and late stages of the race. For optimal 24 h ultra-marathon performance, the “big three” strategies of training, nutrition, and pacing are all important.
Micklewright et al. [39]	34 experienced endurance runners in the Stour Valley Path 100kmUltramarathon	Quasi-Experimental	Risk perception (2 groups), Emotional intelligence(2 groups)	Deviations in average speed from each segments	Each segment-6 splits	Positive	A positive pacing pattern was observed in the ultra-marathon.Perceptions of risk are significantly associated with pacing strategy, with a greater perception of risk were found to adopt a more conservative initial pacing strategy.
Cuk et al. [29]	17,525 finishers in the 2017 Vienna City Marathon	Descriptive	Sex(2 groups), Race (2 groups)	Average speed from each segments, ACSS	5 km-5 splits (half marathon) 10 km-5 splits (full marathon)	Positive	The pacing in half-marathon was more even than in marathon. The more even pacing in women previously observed in marathon races was verified in halfmarathon. The sex difference in pacing was smaller in half-marathon than in marathon.
Tan et al. [14]	167 finishers from Craze Ultra-marathon in Singapore(2012,2013)	Descriptive	Performance Level (3 groups)	Average speed from each segments, Percentage of the mean speed in full race from each segments,End Spurt,CV of speed	17 splits (161kn), 11 splits (101 km)	Parabolic(reverse J-shaped)	Pacing patterns during a 161-km and 101-km ultra-marathon remained consistent across different performance categories.Faster finishers ran with fewer changes in speed than the slower finishers in the 101-km category; finishers remained conservative in their pacing over the last segment of the race.
Andrew et al. [32]	60 finishers in the women's marathon event at the 2009 IAAF World Athletic Championships	Descriptive	Performance Level (4 groups)	Average speed from each segments, Percentage of the mean speed in full race from each segments	5 km-9 splits	Negative	There were differences in pacing strategies displayed by successful and less successful athletes. The most successful athletes achieved finishing times closer to their PBs,
García-Manso et al. [56]	147 elite runners from Frankfurt marathon(2008–2018),Eliud Kipchoge in Monza 2017(Italy) and Vienna 2019(Australia)	Descriptive	NR	Average speed from each segments, Dif-Half	5 km-9 splits	Positive	The race strategy among high-level runners is to maintain a steady speed during the first part to later slightly decrease the speed in the last phase of the race. It seems to be the most effective to achieve an optimal result among runners.
Rodrigues Júnior et al. [30]	10 well-trained marathon runners	Quasi-Experimental	NR	Thermal index, Heart Rate Physical characteristic, Average speed from each segments	3 km-7 splits	Positive	Most of the participants reduced their pace when a T-core of 39 °C was reached which occurred between 6 and 9 km of the half marathon race. However, participants were able to show a slight increase in running speed at 21 km which is consistent with the “end-spurt” phenomenon.
Hernando et al. [62]	88 runners who are 35–40 yr in the 2016 Valencia marathon	Quasi-Experimental	Sex(2 groups)	Physiological characteristics, Relative-Intensity Levels of Physical Activity, Average speed from each segments, ACSS	5 km-9 splits	Positive	Females maintained a more stable pacing and ran at less demanding intensity throughout the marathon, limiting the decay of running pace in the last part of the race.Females run at a more conservative intensity level in the first part of the marathon compared to males).

Note: NR not refer, PB personal best, WR world record.

### 3.1 Marathon categories and characteristics

The 39 included studies investigated the pacing profiles of finishers in half-marathon, full-marathon, and ultramarathon races. Notably, five studies examined both half and full marathons from the same event [17,[26], [27], [28], [29]]. The most frequently studied participants among the 39 studies were full marathon runners (n = 21) and ultramarathon runners (n = 11); conversely, only two studies focused exclusively on the pacing characteristics of half-marathon runners [9,30]. However, no studies explored pacing strategies for half-marathons alone, as five studies provided comparisons between half- and full-marathon pacing characteristics to argue for different pacing strategies. The majority of the studies included both male and female participants (n = 26), 11 studies exclusively included male participants, and only two focused solely on female participants [31,32]. Fifteen studies encompassed all finishers in marathon races, 11 focused on elite or master-level participants, and six on trained participants. World marathon records were addressed in seven studies. Notably, three studies specifically focused on individual ultramarathon athletes [[33], [34], [35]], and one study included a comparison of father and son participants [36].

### 3.2 Main findings

**Fig. 2:**
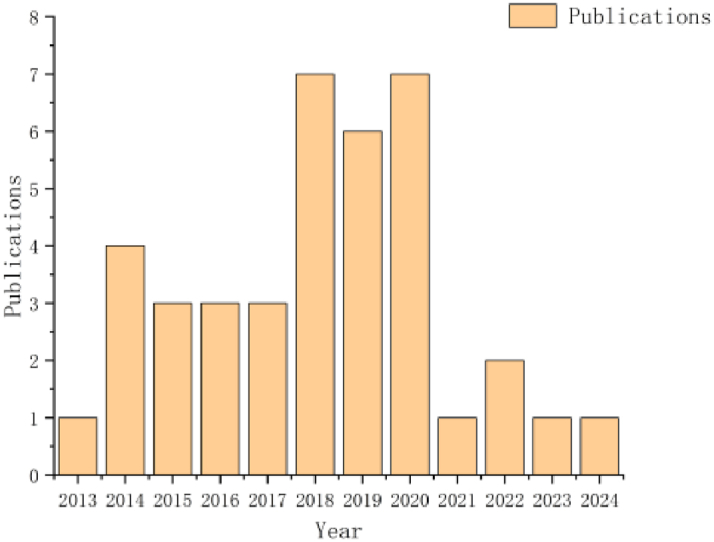
The number of studies examining marathon pacing strategies between 2013 and 2024.

Over the past decade, significant growth has been observed in the analysis of marathon pacing strategies (Fig. 2). Despite a decrease in the number of studies published over the last three years, the investigation of pacing strategies for marathon races likely represents a relatively new research area. A total of 22 studies included speed data from at least 1000 participants, while 17 studies collected speed data from fewer than 1000 runners.

#### 3.2.1 Variables in the evaluating pacing strategy

The variables reported in the studies evaluating pacing strategies are detailed in Table 2. Nearly all studies analyzed the pacing strategies of marathon runners using speed indicators. Absolute speed was measured in a total of 36 studies. Regarding relative speed, 31 studies conducted calculations and utilized relative speed as a principal index for assessing pacing strategies, with 28 studies measuring both relative and absolute speeds. Specifically, the majority of the articles (n = 16) employed a normalized percentage of average race pace, six studies referred to Dif-Half, three studies calculated deviations from average segment speed [[37], [38], [39], [40]], and four studies implemented the ACSS [26,41,42]. Additionally, the Pace Range, End Spurt, and the coefficient of variation of speed were identified as key indicators for evaluating changes in pacing [9,26,36,38,[41], [42], [43], [44], [45], [46], [47], [48], [49], [50], [51], [52]].

**Table 2:** Defining speed variables for pacing strategies in the marathon.

	Speed Variables
1	Average speed in full race and each splits: the mean speed was calculated by dividing split distance by time for each split.
2	The percentage of average change in speed for each segment(ACSS)/average change in speed(ACS): ACSS = 100 − (100 × average segment speed/average race speed),ACS = (ACSS1 + ACSS2 + ACSS3 + ACSS4 + ACSS5)/5.
3	Deviations in average speed from each segments:The deviation of the average speed in the first segment from each segments In fact, it is similar to the ACSS in that one is based on the average speed of the race and the other is based on the average speed of the first split.)
4	Normalized percentage of the mean speed in full race from each segments:100 × (average segment speed/average speed).
5	Pace Range/Pacing Range(PR):The race sections were expressed as a percentage faster or slower than the average section speed. The fastest section for each individual was then named the “positive range” (PR), while the slowest segment was named the “negative range” (NR). In addition, the absolute sum of the positive range and negative range was calculated and named the “pace range” (PR). This method allowed for normalized speed comparisons between all athletes as well as between the marathon and half-marathon.
6	Dif-half:Difference in relative speed between the first half of the marathon and the second half.
7	End Spurt(ES):running speed for the last segment was expressed as a percentage faster or slower than the running speed during former section.
8	Critical Speed(CS):was calculated from the runner's personal best performances in the 3000 m and half marathon.
9	The Coefficient of Variation (CV) of speed: CV =(SD/Mean) × 100

#### 3.2.2 Types of pacing strategies

**Fig. 3:**
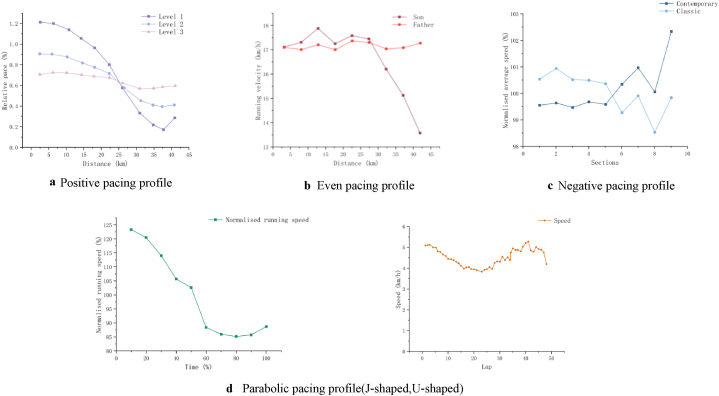
a Positive pacing profile. b Even pacing profile. c Negative pacing profile. d Parabolic pacing profile(J-shaped,U-shaped).

Abbiss and Laursen investigated and described the pacing strategies observed in athletic competitions, categorizing them into six types: negative, all-out, positive, even, parabolic-shaped, and variable pacing strategies [8]. All of the 39 included studies described or defined the pace profiles of marathon runners, referring to the running speed at various stages of the race. Four different pacing strategies were identified (Fig. 3): positive (Fig. 3-a) [17], even (Fig. 3-b) [36], negative (Fig. 3-c) [46], and parabolic (Fig. 3-d) [34,53], with a general aim across studies to achieve an even pace during marathons. Specifically, 22 studies concluded that a positive pacing strategy was employed, two studies indicated that marathon runners maintained even pacing [40,42], one study exclusively identified a negative pacing strategy [54], and three studies reported parabolic-shaped pace profiles [14,34,55]. Notably, eight studies presented two different pacing strategies when comparing different athletes across various marathons, primarily positive, negative, and parabolic pacing [17,31,36,46,52,53,56,57]. Furthermore, two studies explored alternative approaches to pacing strategies, suggesting that marathon runners should either maintain a controllable pacing strategy to intentionally minimize pacing variability [41] or adapt their pacing strategy based on the race environment, thus employing a variable strategy according to optimal pacing principles [37].

#### 3.2.3 Influences on pacing strategies

Almost all studies concur that factors influencing pacing strategy are associated with alterations or interferences in an athlete's split speed. Consequently, when analyzing factors affecting pacing strategy, authors frequently cluster athletes to determine whether those with differing characteristics exhibit variations in speed or changes in speed across each segment and the entire race (absolute or relative). Key factors identified as influencing pacing strategies include gender, age, performance level, marathon category, packing, and physiological/psychological characteristics.

A total of 11 studies considered gender as a subgroup to explore the similarities and differences in the pacing strategies of male and female athletes. Nine studies observed a difference in pacing between men and women, primarily in the magnitude of speed change, with no significant differences in overall profiles. Only two studies reported inconsistent pacing strategies between the genders, with one indicating that men used negative pacing and women did not; the other noted that males decreased their pacing at 25–30 km, while females increased theirs [[27], [28], [29],^41^,^42^,^45^,[47], [48], [49], [50],^57^].

Regarding age, ten studies yielded two main findings about its impact on pacing strategy. Half of these studies suggested that age does not influence an athlete's stable pacing strategy, while the other half indicated that younger and older athletes experience greater decreases in speed. One study, examining the pacing strategy of a world record over 50 years, discovered a shift in strategy, with classic athletes tending toward a more positive pacing and modern athletes a more negative approach [26,28,41,42,[45], [46], [47],^50^,^55^,^58^].

Performance level has been a significant focus in studies of pacing strategies, with all 16 studies affirming that athletes at different performance levels exhibit distinct pacing characteristics. However, these differences are mainly in terms of speed variations, while overall pacing strategies remain relatively consistent. Exceptionally, three studies found that elite runners adopted distinct pacing strategies [31,43,48]. It is generally agreed that the pacing strategies of high-level runners align more closely with the even-rate model, explaining their fewer speed fluctuations compared to other participants [9,14,38,41,42,45,47,51,52,55,59].

Environmental conditions are recognized as significant factors in marathon pacing strategies, with current research focusing on physiological aspects such as body temperature and heat stress [30,45]. Both studies indicated that in warmer temperatures, athletes tend to adopt slower initial speeds to maintain or increase their pace in the latter stages of the race. One study explored the impact of slope and wind on pacing strategies by using ordinary least squares (OLS) regression to estimate the effects of head/tailwinds or slopes on athletes’ pacing, finding that wind significantly affects pacing in the absence of a gradient [37].

Analyses of race categories have currently focused on full and half marathons, with all five studies confirming that the half marathon is more evenly paced and has less pacing variation than the full marathon. However, the end spurt was found on several occasions during the full marathon but not during the half marathon. For the pacing strategy, the full and half marathons were similar to the positive pacing strategy, but sometimes negative pacing was observed in the half marathon [17,[26], [27], [28], [29]].

There are also some studies on the packing behavior of marathon runners; although the number of marathon runners is limited at present (n = 3) [9,52,60], the conclusions thus far have shown that the group will have an effect on the pacing strategy. Other factors, such as individual characteristics, are also widely regarded as factors affecting pacing strategies [30,[33], [34], [35], [36],^39^,^42^,^45^,^61^,^62^]. Two studies demonstrated that fluctuations in blood glucose levels do not relate to pacing strategy, as athletes are capable of maintaining stable blood glucose levels throughout a race [35,61]. Changes in body metabolism are also noted to influence pacing [34,42,62], although the specific reasons for these effects are not uniformly agreed upon and fall into two main theories: one suggests that body temperature and heart rate impact pacing performance [30,36], while the other attributes changes to the dynamics of body weight and composition reaching equilibrium [33]. Additionally, risk awareness influences pacing strategies, with more risk-averse athletes likely to adopt a more conservative starting pace [39]. However, the precise effects of these factors on pacing strategy are not fully elucidated, and scholars often analyze them independently of pacing strategy.

### 3.3 Methodological considerations

Regarding study design and measured pacing variables in the systematic review, Table 3 categorizes the 39 selected studies into two primary study designs: speed and segment. Descriptive and experimental designs are the most commonly employed in pacing strategy research, with descriptive studies comprising 74.4 % of the investigations. In terms of measuring segment speeds, the studies employ various approaches, including the use of absolute and relative speeds, different segment types, and choices of distances, such as the number of laps, time, or the distance of the race to define each segment.

**Table 3:** Methods used in analysing pacing strategies in different study designs.

Study design	Studies	Segment Type			Speed Type			Data Source		Statistical Analysis		
		Distance	Time	Lap	AS	RS	AS/RS	OF	UOF	DA	CA	RA
Descriptive	29	27	2	–	2	2	25	27	2	27	1	1
Quasi-Experimental	10	5	1	4	6	1	3	6	4	6	1	3
Total	39	32	3	4	8	3	28	33	6	33	2	4

Note: AS absolutely speed, RS relatively speed, OF official open website, UOF unofficial data, DA difference analysis, CA correlation analysis, RA regressive analysis.

In marathon research, 28 studies combined absolute and relative speeds to characterize pacing through absolute speeds, describe changes in pacing by measuring relative speeds, and ultimately identify pacing strategies by integrating both types. Predominantly, studies preferred using distance for segmentation due to the accessibility of pace data on official public marathon websites, and because the official split time is recorded by the electronic chip in the athletes’ shoes [36], which activates at set distances [33], thus ensuring data accuracy. Nevertheless, time and lap segmentation, such as using hours [35,55] or laps (400 m/1 km per lap) [33,53], also provide intuitive and reliable data for longer and more duration-intensive events like ultramarathons. Segmentation by the number of laps requires that the race occurs at a circuitous location [51,61], offering its own advantages.

Most studies (n = 33) employed analysis of variance (ANOVA) to test for variability in pacing between or within groups by grouping. Linear regression was more commonly used in experimental studies [34,58], although it is less directly relevant to pacing strategy [63,64]. However, several models have been suggested for predicting pacing performance, highlighting various influencing factors [[65], [66], [67]].

### 3.4 Quality of articles

Table 4 shows the NOS results for the quality of the included articles. The evaluation of article quality indicated a median NOS score of 8, with a range of 5–9. The median scores were reported as follows: 3 for ‘selection’ (range 2–4), 2 for ‘comparability’ (range 1–2), and 3 for ‘outcome’ (range 2–3). About 30 % of the studies (n = 12) achieved a maximum score of 9. According to the NOS, 34 articles were deemed ‘good’ (scale 7–9), and five were considered ‘fair’ (scale 4–6) [14,30,46,49,53]. It was reported that no included article disclosed a potential conflict of interest; 26 articles declared no conflict of interest, whereas 13 articles omitted reporting on potential conflicts of interest.

**Table 4:** Quality assessment based on the Newcastle–Ottawa Scale (NOS) of articles in the systematic review.

Article	Selection (/4)	Comparability (/2)	Outcome (/3)	Total (/9)
Cuk et al. [26]	4	2	3	9
Louis et al. [36]	3	2	2	7
Sengoku et al. [61]	3	1	3	7
Muñoz-Pérez et al. [43]	4	1	3	8
Angus et al. [37]	2	2	3	7
Nikolaidis et al. [38]	4	2	3	9
Arie-Willem et al. [17]	4	2	2	8
Trubee et al. [45]	4	1	3	8
Oficial-Casado et al. [44]	4	2	3	9
Matta et al. [53]	2	1	3	6
Andrew et al. [32]	3	1	3	7
JoJo et al. [46]	2	1	3	6
Andrew et al. [47]	3	2	3	8
Santos-Lozano et al. [48]	4	2	3	9
Breen et al. [41]	4	2	3	9
Beal et al. [31]	3	2	3	8
JoJo et al. [49]	2	1	3	6
Knechtle et al. [33]	3	2	2	7
Knechtle et al. [34]	3	2	3	8
Nikolaidis et al. [50]	4	2	3	9
Nikolaidis et al. [27]	3	2	3	8
Brian Hanley [9]	4	2	3	9
Nikolaidis et al. [42]	3	2	3	8
Aschmann et al. [40]	4	1	3	8
Bossi et al. [55]	3	2	3	8
Takayama et al. [51]	3	1	3	7
Knechtle et al. [58]	3	2	3	8
Brian Hanley [52]	4	2	3	9
Nikolaidis et al. [28]	4	2	3	9
Billat et al. [54]	3	1	3	7
Takayama et al. [35]	3	2	3	8
Micklewright et al. [39]	3	1	3	7
Cuk et al. [29]	4	2	3	9
Tan et al. [14]	3	1	2	6
García‐Manso et al. [56]	3	1	3	7
Rodrigues Junior et al. [30]	2	1	2	5
Hernando et al. [62]	4	2	3	9
Arturo et al. [57]	3	2	3	8
Muñoz-Pérez et al. [60]	4	2	3	9
Median (range)	3 (2–4)	2 (1–2)	3 (2–3)	8 (5–9)

## 4 Discussion

### 4.1 Pacing profile and pace range: reflection of the marathon pacing strategy

In marathon running, various methods exist to describe an athlete's pacing strategy, primarily through pacing profiles and pacing ranges. Pacing profiles, by generating curves from different data segments, visually depict and analyze pacing trends. Typically, data segmentation involves either absolute or relative velocities. Absolute speeds facilitate direct comparisons of pacing profiles among athletes and the analysis of variations in their running speeds [30,[34], [35], [36],^61^,^68^]. In contrast, relative speed more accurately reflects an athlete's individual stride speed trends. Unlike absolute speed, relative speed does not enable effective comparison between different athletes [17]. However, segmenting average speeds allows a clearer understanding of individual pace changes in athletes [37,39]. Most articles utilize nine data segments for plotting at distances of 5, 10, 15, 20, 25, 30, 35, 40, and 42.195 km [38,46,49], aligning with the nine official subsites in competitions [45]. This segmentation ensures accuracy in data capture, covering nearly all athletes' pacing data available online, ideal for research [48], and minimizing data collection errors [62]. Nevertheless, this method's less frequent segmentation [50] might obscure finer details in pacing curves, hindering a comprehensive analysis of a runner's pacing.

Pace range, often overlooked, emerges as a crucial metric in pacing strategy studies. It is insufficient to define pacing strategies solely through pacing profiles, yet this error recurs in numerous publications. Thus, scholars have introduced the concept of pace range to provide a simple, macroscopic view of extreme variance in pacing [26,42], facilitating direct comparisons and highlighting different pacing characteristics during a race [28,41]. Unlike a uniform approach, some researchers have compared and standardized average speed ratios from the race's first and second halves to distinguish between even and uneven pacing, defined as negative or positive [9,44,52,56]. For example, Iker et al. set a criterion of 10 percent to categorize pacing as uneven if the time ratios in the first and second halves of the race exceed this mark, and as even if they do not [43]. Robert et al. calculated the percentage change in velocity observed in the second half of the marathon compared to the velocity in the first half, referring to this as pace maintenance [69]. Percentage changes of less than 10 % were classified as “maintaining the pace,” while percentage changes greater than 30 % were classified as “marked slowing.” Therefore, a uniform criterion is needed to better assess the pacing strategy.

### 4.2 Changing trends in pacing strategy: the evolution of marathon races

Athletes' pacing strategies adapt to varying race conditions in a marathon, an open outdoor event susceptible to fitness loss, unfavorable positioning, and environmental changes [70,71]. As high-level competitors, athletes continuously adjust their pacing based on current conditions to optimize their race performance without compromise [39,72]. Billat et al. observed such adjustments in Eliud Kipchoge's races, noting that even world-class athletes modify their pacing strategies to set new world records [54]. These adjustments are also evident in systematic training that allows athletes to improve race times [16,35,73]. JOSÉ JOAQUÍN et al. noted a shift in world record pacing strategies over the past 50 years, with a prevailing trend towards more even pacing [46]. Despite these shifts, analyzing each race's pacing strategies remains a key method for understanding current trends and exploring optimal pacing strategies [37].

### 4.3 Inconsistent pacing strategies: gender differences among athletes

Gender differences are frequently discussed in sports, notably in marathons. Male and female athletes exhibit distinct preferences in pacing strategies [49]. Males typically adopt an aggressive pacing strategy, characterized by a rapid start and a speed peak between 10 and 25 km, followed by a decline in speed, whereas females generally choose a more conservative strategy, starting slower and maintaining a stable pace in the initial race stages, with a gradual acceleration post the 25 km mark [45,50]. These differences are attributed to psychological factors influenced by gender [42]. Men, possessing greater confidence and higher performance expectations, often opt for a quicker initial pace. In contrast, women, focusing more on race completion rather than early positioning, prefer to ensure race completion without overextending early [48,52]. This gender disparity extends beyond pre-race strategy. Robert et al. observed that male athletes experience a more significant speed reduction during races compared to female athletes, likely due to higher initial energy expenditure [69,74]. Both psychological motives, such as competitive early pacing [69], and physiological aspects, such as nerve fatigue and muscle glycogen depletion, influence these patterns [75]. Men, typically exhibiting poorer fat utilization, are more prone to deplete muscle glycogen stores compared to women, who may have more efficient fat metabolism [76,77]. Female athletes tend to display a more uniform and consistent pacing strategy, potentially making it easier to identify optimal pacing strategies for them [27].

### 4.4 The impact of distance: the persistent influence on the pacing strategy

Marathons, categorized into half-marathons (21.0975 km), full marathons (42.195 km), and ultramarathons (exceeding 42.195 km), demonstrate varied pacing strategies across distances, with consensus in the literature on this variability [[26], [27], [28]]. The nature of the event, relying solely on running, places physical fitness as a crucial determinant of pacing [41]. In half-marathons, athletes might initiate an aggressive pace due to the shorter remaining distance, allowing for a sustained, high-intensity effort toward the end [29,78]. Conversely, early acceleration in a full marathon can lead to substantial deceleration in the latter stages [79]. Ultramarathon participants often adopt a conservative pacing strategy, maintaining minimal variation between their peak and average paces throughout the race [14,34,80]. Additionally, the distance not only impacts energy distribution but also significantly influences training costs. Swain et al. analyzed training across various marathon events and found no substantial differences in training duration or race experience among athletes, though pacing strategies varied [16]. The requirements for shorter events demand more intensive training relative to longer events, facilitating a more effective strategy discovery for athletes.

### 4.5 Simplifying pacing strategies: the quest for three ideal approaches in the marathon

Contrary to Abbiss and Laursen's categorization of six pacing strategies [8], this study posits that only three pacing strategies are prevalent in marathons: positive, negative, and even. This assertion stems from the subjective nature of pacing profile measurements in existing literature, which often relies on the discretion of researchers [14,26,36,41,54,55,58,61]. The terms “positive," “negative,” and “even” emerge as the most frequently used descriptors in the analyzed studies [27,29,42,46,49]. While some research suggests parabolic pacing strategies [1,34,48], these are essentially nuances within the broader categories of positive or negative pacing. Therefore, it is argued that marathon pacing strategies should be simplified into these three fundamental types. Further analysis of pacing, such as identifying parabolic or fluctuating trends, should be considered from a more detailed, microscopic viewpoint.

### 4.6 Quality of evidence

The review of 39 articles revealed a high quality of research, although five articles scored below 7 on the NOS scale. Notably, only about half of the studies utilized representative samples from various races, indicating a need for a more diverse sample selection in future studies. The ‘comparability’ component showed the greatest variability, influenced by differences in reported variables such as pacing metrics, training characteristics, and athlete levels. Enhancing methodological rigor in these areas is crucial for future research.

### 4.7 Implication and future research

This study presents significant practical implications for athletes and coaches. It is suggested that coaches could devise targeted training programs to assist athletes in achieving the requisite fitness for acceleration, deceleration, and pace maintenance at different stages of a race. This information could serve as a reference point for athletes to adopt a strategic pacing plan to navigate the various stages of a race and secure an optimal finish time. Moreover, the comprehension of different runners’ pacing strategies enables better preparation and adaptation to varying styles of opponents. Such knowledge is instrumental in developing suitable tactics and pacing that enhance the likelihood of victory in a race. Additionally, an examination of pacing strategies in marathon races offers a comprehensive macro- and micro-perspective on RP, proving extremely beneficial for scholars. This contributes to a better understanding of the variations in physiological and psychological characteristics of athletes.

The current method permits only the analysis of extended subparagraphs in marathons (5 km/10 km), and it remains uncertain whether a more distinct change in runners’ pacing at higher frequency segments would facilitate a clearer differentiation of pacing strategies. In marathons, terms like “hitting the wall (HTW)”, “bonking”, or “blowing up” describe the sudden emergence of debilitating fatigue that can occur late in the race. At best, this may temporarily decelerate even the most skilled and experienced runners, but it may also limit a runner to little more than a walking pace for the remaining race duration and could prevent some from finishing [71]. Therefore, “the wall”, as a specific phenomenon in marathons, has a significant impact on the speed of runners in the latter stages of the race, and it remains an unresolved issue how much it will alter the pacing strategy [70]. For marathon runners, additional physiological and running parameters such as step length/frequency [81], heart rate [82], maximum oxygen uptake, energy cost of running, and running economy [83,84] need to be considered to optimize their performance over extended periods and distances [85]. Training should also be aligned with runners to comprehend the volume and intensity of training. This finding aligns with their performance pacing in races to enhance the understanding of their pacing strategies.

### 4.8 Strengths and limitations

This study offers the initial overview of pacing strategies in marathon races, and unlike prior qualitative analyses of such pacing behaviors, this paper provides a quantitative analysis from the perspective of segmented pacing data. Not only have the pacing strategies currently observed in marathons been summarized and categorized, but an attempt has also been made to understand the pacing features of marathons at both the macro and micro levels.

The application of the NOS, a quality assessment tool endorsed by the Cochrane Collaboration, bolsters the methodology of this review. This is further supported by the involvement of a second author to validate the results of the scale, as the interrater reliability of the NOS has been critically examined [86]. However, the sensitivity of the NOS might be questioned. For example, the first question of the scale concerning the representativeness of the exposed cohort, and both options (a) ‘truly representative’ and (b) ‘somewhat representative’ receiving the same score, represents a limitation of the scale.

Although this review was conducted in accordance with the PRISMA guidelines updated in 2020 and involved standardized critical appraisal, it still possesses some limitations. The primary limitation is that only English-language articles were retrieved. Additionally, there are currently limited tools and instruments available for collecting detailed segmental pacing data in marathon races. Furthermore, the diverse range of data sources and metrics included in this review hinders the establishment of a standardized framework for evaluating pacing strategies. Consequently, the findings depend more on a qualitative, contextual assessment rather than quantitative comparisons and benchmarks. Hence, future studies should focus more on the pacing performance of athletes in the high-frequency segments of the race.

## 5 Conclusion

This systematic review synthesized the available pacing data collected during competition from marathon runners to quantitatively analyze and understand pacing strategies. Pacing strategy is defined as a runner's performance across the various phases of the race. It was found that: (1) pacing profiles and ranges are the primary manifestations of the pacing strategy; (2) the pacing strategy is subject to continuous change and is significantly influenced by gender and distance; and (3) there appear to be only three types of pacing strategies for marathons: positive, negative, and even. Factors such as pack dynamics, physiological and psychological characteristics also potentially influence marathon pacing and require further analysis and exploration. Moreover, the limiting factors of the pacing strategy are primarily focused on segmentation frequency. Therefore, an increased frequency of segmentation could provide better insights into the microlevels of pacing strategies and suggest more effective timing concepts and training programs for marathon runners and coaches to rationally choose the optimal pacing pattern to enhance performance.

## Funding statement

This research did not receive any specific grant from funding agencies in the public, commercial, or not-for-profit sectors.

## Data availability

Data will be made available on request.

## CRediT authorship contribution statement

**Jungong Sha:** Writing – original draft, Visualization, Methodology, Formal analysis, Data curation, Conceptualization. **Qing Yi:** Writing – review & editing, Methodology, Formal analysis. **Xin Jiang:** Resources, Investigation, Data curation. **Zhengwei Wang:** Writing – review & editing, Data curation. **Houwen Cao:** Writing – review & editing, Data curation. **Shan Jiang:** Writing – review & editing, Visualization, Supervision, Resources, Investigation, Conceptualization.

## Declaration of competing interest

The authors declare that they have no known competing financial interests or personal relationships that could have appeared to influence the work reported in this paper.
